# Editorial: The punctual night knocker: circadian rhythm of restless legs syndrome

**DOI:** 10.3389/fneur.2023.1358722

**Published:** 2024-01-08

**Authors:** Xiao-Ying Zhu, Diego Garcia-Borreguero, Yu-Qing Li, Li-San Zhang, Xin-Rong He, Jing Zhang, Dong-Yan Wu, Yun-Cheng Wu

**Affiliations:** ^1^Department of Neurology, Shanghai General Hospital, Shanghai Jiao Tong University School of Medicine, Shanghai, China; ^2^Sleep Research Institute, Madrid, Spain; ^3^Department of Neurology and Neuroscience, Norman Fixel Institute for Neurological Diseases, College of Medicine, University of Florida, Gainesville, FL, United States; ^4^Department of Neurobiology, Center for Sleep Medicine of Sir Run Run Shaw Hospital, Zhejiang University School of Medicine, Hangzhou, China; ^5^Department of Neurology, Center for Sleep Medicine of Sir Run Run Shaw Hospital, Zhejiang University School of Medicine, Hangzhou, China; ^6^Department of Neurology, Hua Shan Hospital, Fudan University, Shanghai, China

**Keywords:** restless legs syndrome, circadian rhythm, sleep, periodic limb movements in sleep, dopamine, iron

Restless legs syndrome (RLS) is a common neurologic sleep-related movement disorder characterized by an urge to move the legs, usually accompanied by uncomfortable sensations localized deep in the lower limbs. Although the exact etiology of RLS is still under discussion (with genetic factors, brain iron deficiency, and various neurotransmitter disturbances in the basal ganglia playing a major role), RLS presents the characteristic identical symptomatology whether primary or comorbid RLS. Five criteria that empirically based upon clinical features are obligatory for RLS diagnosis (1): (1) “urge to move” the legs often accompanied by uncomfortable leg sensations; (2) usually worst at rest (sitting or lying down); (3) usually relieved (although often temporarily) by the movement of the affected limb (i.e., walking or stretching or bending the legs); (4) evident circadian fluctuations with sensory symptoms and motor activities most pronounced in the evening or at night disrupting sleep; and (5) exclusion of RLS mimics. Except the last exclusion criteria, the first four criteria describes four distinct aspects of RLS, including the 4^th^ circadian criteria. Walters and Zee reviewed the point that criteria 2 (immobility) and 4 (circadian rhythmicity) were related but different separate phenomena. Immobility (typically while sitting or lying down) worsens RLS symptoms at any fixed time of the day which confirms criteria 2. However, the effects of immobility to RLS symptoms is subject to circadian modulation. Furthermore, both the sensory and motor aspects of RLS show a similar diurnal pattern that worsens at night independent of body position, which confirms the separate role of criteria 4. A total of 80% RLS patients are comorbid with periodic limb movements in sleep (PLMS). PLMS also demonstrate an evident circadian pattern and are considered a significant marker of RLS. Woods et al. described that PLMS occurred mostly during stages 1 and 2 of NREM sleep, at the beginning of the main sleep period.

RLS is regarded as a complex sensorimotor network disorder with enhanced excitability and/or decreased inhibition involving cortical, subcortical, spinal cord, and possible peripheral nerves. The hyperexcitability of RLS neural network is modulated by the circadian influence, although the exact cause of RLS is unknown (2). Genetic and environmental factors are considered to contribute to RLS development. Brain iron deficiency, central dopaminergic dysfunction, together with the glutamate, opiate, and adenosine system disturbances, are suggested to play essential roles in RLS pathophysiology, causing increased CNS excitability with characteristic diurnal variation. Lines of evidence suggest spinal cord excitability in patients with RLS and PMLS (3). However, little has investigated if iron deficiency correlates with spinal hyperexcitability. Woods et al. in their article demonstrated that an iron deficiency diet could induce reversible RLS-like symptoms in mice with regard to sleep onset and spinal cord reflex excitability, suggesting iron deficiency also had an impact on spinal hyperexcitability besides the brain. They therefore raised the suggestion to use the term “iron deficiency in the central nervous system” (ID-CNS) instead of the commonly used phrase “brain iron deficiency” (BID), to include possible effects of altered iron homeostasis on spinal cord function. Woods' study also indicated that a dysregulation of iron metabolism affects sleep-wake cycle and especially influence the onset of sleep, a pattern similar to RLS that predominantly occurred at the beginning of sleep episodes in stages 1 and 2 of NREM sleep.

Supraoptic nucleus locates within the hypothalamus mediates the circadian clock. As a clear circadian feature of RLS, the role of circadian clock and supraoptic nucleus in RLS needs further investigation. Tang et al. in their article reviewed the role of circadian clock and supraoptic nucleus in regulating RLS. They summarized series of cases, including a RLS case with severe delayed sleep–wake phase disorder (DSWPD), cases in shift workers, as well as RLS patients with cross-temporal flights, whose RLS symptoms occurred in a phase of either delay or advance that coincided with the time before sleep onset. These cases suggest that RLS symptoms are modulated by endogenous circadian rhythm. Tang et al. also described the diurnal variation of RLS symptoms significantly associated with the core body temperature cycle, and the most prominent symptoms corresponding to the nadir of the core body temperature variation, suggesting circadian mechanisms in RLS regulation. Such modulation might not be the result of a direct action of the endogenous pacemaker, because its function has shown to remain intact in RLS patients. Moreover, the time of onset of RLS symptoms seems to be the result of indirect changes caused upon the circadian clock, affecting iron metabolism, melatonin, and other factors. Interestingly, dopaminergic drugs show phase-advancing effects on the Dim Light Melatonin Onset (DLMO) (4).

Augmentation is frequent in RLS patients with long-term dopaminergic treatment. Interestingly, one of the main features of augmentation is a phase advance with anticipated initiation of RLS symptoms. Dopaminergic agents might lead to this treatment complication by acting on the circadian pacemaker due to its phase-advancing effects on the DLMO (4). Woods et al. and Zeng et al. in their article mentioned that DLMO, a marker of the endogenous circadian phase, was found to occur earlier in RLS patients undergoing augmentation, corresponding to an earlier onset of RLS symptom with augmentation (4). Zeng et al. reviewed in their article that multiple factors may induce augmentation in RLS, and BID/ID-CNS is also a critical factor contributing to RLS augmentation, which may exacerbate RLS symptoms by affecting the function of the dopamine system. Zeng et al. and Tang et al. reviewed in detail on the diurnal rhythmicity of iron concentration and the dopamine system dynamics, and how they work together.

Both circadian and homeostatic mechanisms regulate sleep. Criterion 4 of IRLSSG's criteria highlights a clear circadian feature of RLS, which is separate from other criteria including immobility. It has been suggested that various neural centers located in the brainstem and underlying dopaminergic control might be involved: As such, a decrease of sensory input caused by active movement or by sensory stimulation would ultimately activate these “gate control” centers in the brainstem and alleviate RLS symptoms (5). As a complex sensorimotor network disorder, RLS presents multiple levels of CNS hyperexcitability including the spinal cord. Iron deficiency can exacerbate this process of neural hyperexcitability. RLS presentations in DSWPD, shift workers, and during cross-temporal flights, as well as endogenous circadian markers like core body temperature cycle and DLMO that related to RLS symptoms, together suggesting circadian mechanisms in RLS pathogenesis. The detailed molecular mechanisms on RLS circadian regulation need further investigation.

## Author contributions

X-YZ: Conceptualization, Project administration, Writing – original draft, Writing – review & editing. DG-B: Conceptualization, Writing – review & editing. Y-QL: Conceptualization, Writing – review & editing. L-SZ: Conceptualization, Writing – review & editing. X-RH: Writing – review & editing, Investigation. JZ: Writing – review & editing, Investigation. D-YW: Writing – review & editing. Y-CW: Supervision, Writing – review & editing.
